# Genetic testing in inherited endocrine disorders: joint position paper of the European reference network on rare endocrine conditions (Endo-ERN)

**DOI:** 10.1186/s13023-020-01420-w

**Published:** 2020-06-08

**Authors:** Thomas Eggermann, Miriam Elbracht, Ingo Kurth, Anders Juul, Trine Holm Johannsen, Irène Netchine, George Mastorakos, Gudmundur Johannsson, Thomas J. Musholt, Martin Zenker, Dirk Prawitt, Alberto M. Pereira, Olaf Hiort, on behalf of the European Reference Network on Rare Endocrine Conditions (ENDO-ERN, Stefan Riedl, Birgit Rami-Merhar, Greisa Vila, Sabina Baumgartner-Parzner, Walter Bonfig, Claudine Heinrichs, Dominique Maiter, Inge Gies, Martine Cools, Kristina Casteels, Albert Beckers, Sabina Zacharieva, Violeta Iotova, Tomislav Jukic, Dario Rahelic, Vassos Neocleous, Leonidas Phylactou, Michal Krsek, Jan Lebl, Claus Gravholt, Anders Juul, Vallo Tillmann, Vallo Volke, Tapani Ebeling, Thierry Brue, Patrice Rodien, Jérôme Bertherat, Christine Poitou Bernert, Philippe Touraine, Philippe Chanson, Michel Polak, Maithe Tauber, Thomas Eggermann, Joachim Spranger, Dagmar Fuhrer, Thomas Danne, Olaf Hiort, Klaus Mohnike, Dirk Prawitt, Markus Luster, Nicole Reisch, Martin Reincke, Julia Rohayem, Martin Fassnacht, Miklós Tóth, Alessandra Cassio, Sonia Toni, Csilla Krausz, Barbara Piccini, Diego Ferone, Gianni Russo, Luca Persani, Annamaria Colao, Mariacarolina Salerno, Marco Boscaro, Carla Scaroni, Ferruccio Santini, Giovanni Ceccarini, Ezio Ghigo, Iveta Dzivite - Krisane, Vita Rovite, Lauma Janozola, Rasa Verkauskiene, Michael Witsch, James Clark, Johannes Romijn, Thera Links, Nienke Biermasz, Sabine Hannema, Bas Havekes, Hedi Claahsen-van der Grinten, Henri Timmers, Robin Peeters, Gerlof Valk, A. A. Verrijn Stuart, Harm Haak, Eystein Husebye, Jens Bollerslev, Barbara Jarzab, Agnieszka ‘Szypowska, João-Filipe Raposo, Dana Craiu, Doina Piciu, Ludmila Kostalova, Jarmila Vojtková, Tadej Battelino, Roque Cardona-Hernandez, Diego Yeste, Sonia Gaztambide, Anna Nordenström, Neil Gittoes, Trevor Cole, Elizabeth Crowne, Faisal Ahmed, Mohammed Didi, Marta Korbonits, Mehul Dattani, Peter Clayton, Justin Davies

**Affiliations:** 1 Institute of Human Genetics, Medical Faculty, RWTH Aachen, Pauwelsstr. 30, 52074 Aachen, Germany; 2 Department of Growth and Reproduction, Rigshospitalet, University Hospital of Copenhagen, Copenhagen, Denmark; 3 International Center for Research and Research Training in Endocrine Disruption of Male Reproduction and Child Health (EDMaRC), Rigshospitalet, University of Copenhagen, Copenhagen, Denmark; 4 INSERM, Centre de Recherche Saint-Antoine, Sorbonne Université, UFR Médecine, AP-HP, Hôpital Armand Trousseau-Explorations Fonctionnelles Endocriniennes, Paris, France; 5 Unit of Endocrinology, Diabetes Mellitus and Metabolism, ARETAIEION Hospital, Faculty of Medicine, National and Kapodistrian University of Athens, Athens, Greece; 6 Department of Internal Medicine and Clinical Nutrition, Institute of Medicine, Sahlgrenska Academy, University of Gothenburg and Department of Endocrinology at Sahlgrenska University Hospital, Gothenburg, Sweden; 7 Section of Endocrine Surgery, Department of General, Visceral and Transplantation Surgery, Johannes Gutenberg University Medical Center, Mainz, Germany; 8 Institute of Human Genetics, Otto-von-Guericke-Universität Magdeburg, Magdeburg, Germany; 9 Center for Pediatrics and Adolescent Medicine, Johannes Gutenberg University Medical Center, Mainz, Germany; 10 Department of Medicine, Division of Endocrinology, Leiden University Medical Center, Leiden, the Netherlands; 11 Department of Paediatrics and Adolescent Medicine, Division of Paediatric Endocrinology and Diabetes, University of Lübeck, Lübeck, Germany

**Keywords:** Rare endocrine conditions, Genetic testing, Imprinting disorders, Short stature - glucose and insulin homeostasis - Hypogonadotropic hypogonadism - differences/disorders of sex development

## Abstract

**Background:**

With the development of molecular high-throughput assays (i.e. next generation sequencing), the knowledge on the contribution of genetic and epigenetic alterations to the etiology of inherited endocrine disorders has massively expanded. However, the rapid implementation of these new molecular tools in the diagnostic settings makes the interpretation of diagnostic data increasingly complex.

**Main body:**

This joint paper of the ENDO-ERN members aims to overview chances, challenges, limitations and relevance of comprehensive genetic diagnostic testing in rare endocrine conditions in order to achieve an early molecular diagnosis. This early diagnosis of a genetically based endocrine disorder contributes to a precise management and helps the patients and their families in their self-determined planning of life. Furthermore, the identification of a causative (epi)genetic alteration allows an accurate prognosis of recurrence risks for family planning as the basis of genetic counselling. Asymptomatic carriers of pathogenic variants can be identified, and prenatal testing might be offered, where appropriate.

**Conclusions:**

The decision on genetic testing in the diagnostic workup of endocrine disorders should be based on their appropriateness to reliably detect the disease-causing and –modifying mutation, their informational value, and cost-effectiveness. The future assessment of data from different *omic* approaches should be embedded in interdisciplinary discussions using all available clinical and molecular data.

## Background

Genetic disorders compose a substantial fraction of human diseases, and it is estimated that nearly 5% of live births have a genetically driven illness recognizable until the 25th year of life [1]. However, it is a challenge to diagnose these rare conditions by assessing clinical features and conventional diagnostic testing alone. As a result, many patients and their families undergo a long-lasting diagnostic odyssey.

In clinical endocrine practice, genetic testing is primarily requested to confirm a suspected clinical and endocrine diagnosis, in particular in case the clinical features are ambiguous. Additionally, it also contributes to the identification of presymptomatic individuals. Thereby, their risk to develop an inherited endocrine disorder can be predicted, and prophylactic measures might be taken (i.e. thyroidectomy in carriers of specific *MEN2* variants). This risk can also be determined in relatives of mutation carriers, and the knowledge on an inherited genetic variant is the basis to advice the patients´ family in respect of family planning and prenatal testing. Finally, the precise determination of the molecular alteration causing the endocrine disorder allows to understand its pathophysiology and thereby to develop and apply an adapted therapy.

In recent years, high throughput genetic tests (i.e. next generation sequencing (NGS)) have become increasingly available for clinical use at reasonable costs, and significant progress has been achieved regarding the detection rate in human genetic diagnostic testing [2, 3]. As a result, a diagnostic yield of up to 40% can currently been achieved in genetic heterogeneous disorders, depending on the precision of the clinical assessment and the disorder itself (e.g. [4], for general review: [5]). Molecular alterations also play a major role in tumor development, and NGS has turned out to be an appropriate tool (for review: [6]) for tumor profiling as the basis for treatment and prognosis. Accordingly, genetic testing has become an indispensable component of the comprehensive diagnostic workup in pediatric endocrinology, and increasingly also as part of adult endocrine diagnostics (Table 1), in addition to the common biochemical laboratory analysis. Consequently, the demand for genetic testing continues to increase, and the physician asking for genetic analysis should be aware of the indications for testing, of the used methods and their chances of success, but also of their limitations.

**Table 1:** Genetic testing strategies available for selected endocrine disorders. The disorders are listed according to the main thematic groups of the ENDO-ERN, but there is of course an overlap between them. As it can be deduced from the different examples, the decision about the genetic testing strategies (*) are mainly based on the spectrum of molecular variants and the clinical findings; In disorders, in which NGS-based multigene panel is the most efficient diagnostic testing procedure, this method listed in bold face. However, the listed procedures only represent examples and/or suggestions, but might differ between different laboratories. For further description of methods see Table 2. The four types of molecular changes (**) which can be detected by molecular testing are indicated for the different diseases, but it should be noted that the majority of variants are SNVs. Mode of inheritances (***) are divers, even within the same gene and disorder. In case of autosomal dominant (AD) inheritance de-novo occurrence is frequent

Acronym	Disorder	Gene / Chromosomal Region	OMIM	Genetic testing strategy*	Detection on different molecular levels (rates if available)**				Differential diagnosis	Mode of inheritance***
					SNVs	gene/exon targeted CNV analysis	CNVs	Epimut UPDs		
Genetic adrenal disorders*										
ACC	Adrenocortical carcinoma	*TP53*	#202300	(1. sequencing of specific exons) **2. multigene panel**	yes	yes			*ADCC* can be observed in Beckwith-Wiedemann syndrome (see below) and is a component tumor in Li-Fraumeni syndrome.	AD
APS1	autoimmune polyendocrine syndrome type 1	*AIRE*	#240300	1. single gene testing **2. multigene panel**	yes	yes			Overlap with several disorders.	AR, AD
CNC	Carney complex	*PRKAR1A*	#160980 (type 1)	1. single gene testing 2. CNV analyses 3. multigene panel	60%	10%			Broad clinical spectrum and overlap with several disorders. It includes Cushing syndrome.	AD
PPNAD	Primary pigmented nodular adrenocortical disease type 1	*PRKAR1A*	#610489							AD
	Primary pigmented nodular adrenocortical disease type 2	*PDE11A*	#610475		yes					AD
	Primary pigmented nodular adrenocortical disease type 3	*PDE8B*	#614190		yes					AD
21-OHD-CAH	21-Hydroxylase-Deficient Congenital Adrenal Hyperplasia	*CYP21A2*	#201910	1. single gene testing. CNV analysis	70–80%	20–30%			Major type of CAH.	
Calcium and Phosphate Homeostasis*										
HRPT	Hyperparathyroidism	*CDC73*	#145000	**Multigene panel**	yes	yes				AD
	Neonatal Hyperparathyroidism	*CASR*	#239200		yes					AD, AR
	Familial Isolated Hypoparathyroidism	*GCM2*	#146200		yes					AD, AR
	hypocalciuric hypercalcaemia	*CASR* *GNA11* *AP2S21*	#601198 #145981 #600740		yes					AD
PHP / iPPSD	Pseudohypoparathyroidism / Inactivated PTH/PTHrP Signalling Disorder	*GNAS*	#166350 #103580 #603233 #612462 #612463	Methylation-specific test single gene testing CNV analyses	yes	yes		yes	Heterogeneous group of disorders caused by molecular changes of the imprinted *GNAS* locus.	AD
ADHR	Autosomal dominant hypophosphatemic rickets	*FGF23*	#193100	single gene testing	yes	yes				AD
XLHR	X-linked dominant hypophosaphatemic rickets	*PHEX*	#307800	single gene testing	yes	yes				X-linked
Genetic Pituitary Hormone Disorders*										
CPHD	Combined Pituitary Hormone Deficiency	*PROP1*	#262600	(1. single gene testing) **2. multigene panel**	yes	yes			The diagnosis of combined pituitary hormone deficiency (CPHD) requires the presence of growth hormone (GH) deficiency and deficiency of at least one other pituitary hormone.	AR, AD
		*POU1F1*	#613038							
		*HESX1*	#182230							
		*others*								
FIPA	Familial Isolated Pituitary Adenoma	*AIP*	#102200	single gene testing	yes				Overlap with MEN1	AD, somatic mosaicism
Genetic Thyroid Disorders*										
HCNG	Congenital non-goitrous hypothyroidism	*TSHR*	#275200	**multigene panel**	yes	yes			Molecularly heterogenous group of disorders.	AD, AR
		*SLC5A5*	#274400							
		*PAX8*	#218700							
		*others*								
Glucose and Insulin Homeostasis*										
MODY	Maturity-Onset Diabetes of the Young type 1	*HNF1A*	#600496	(1. single gene testing) **2. multigene panel** 3. CNV analyses	yes	yes			Currently 11 loci for MODY have been identified. 30–65% of patients carry mutations in *HNF1A*, 30–50% in GCK, 5–10% in *HNF4A*.	AD
	Maturity-Onset Diabetes of the Young type 2	*GCK*	#125851							
	Maturity-Onset Diabetes of the Young type 1	*HNF4A*	#125850							
TNDM	Transient neonatal diabetes mellitus	6q24 (PLAG1)	#601410	1. Methylation-specific test 2. single gene testing or multigene panel	no	yes	yes	yes	TNDM accounts for ~ 50% neonatal diabetes. Other genetic causes include pathogenic variants in KCNJ11 and ABCC8 (see PNDM).	sporadic, AD, paternal inheritance; somatic mosaicism
		KCNJ11	#610582		yes					AD
		ABCC8	#610374							;
PNDM	Permanent neonatal diabetes mellitus	*KCNJ11*	#606176	**multigene panel**	yes				*KCNJ11* mutations account for 30% of patients, *INS* 20% and *ABCC8* 19%.J55	AD, AR
		*ABCC8*								
		*GCK*								
		*INS*								
		*PDX1*								
HHF / CHI	Familial hyperinsulinemic hypoglycemia / congenital hyperinsulinism	*ABCC8*	#256450	(1. single gene testing) **2. multigene panel**	yes	yes		UPD as somatic event in focal type	*ABCC8* mutations account for 40–45% of patients. Focal type is due to a paternally inherited *ABCC8* or *KCNJ11* mutation plus somatic loss of heterozygosity (LOH).	AD, AR
		*KCNJ11*	#601820							
		*others*								
Genetic Endocrine Tumour Entities*										
MEN1	Multiple endocrine neoplasia type 1	*MEN1*	#131100	1. single gene testing 2. CNV detection 3. multigene panel	familial: 80–90% single: 65%	1–4%			multigene testing after *MEN1* analysis: *RET, CDKN1B, AIP, CASR, CDC73.*	AD
MEN2	Multiple endocrine neoplasia type 2	*RET*	#171400	1. testing for specific variants (C634R) 2. sequencing of whole gene	98 > 98%					AD
MEN3	Multiple endocrine neoplasia type 3		#162300	1. testing for specific variants (M918T) 2. sequencing of whole gene	98 > 98%					AD
MEN4	Multiple endocrine neoplasia type 4	*CDKN1B*	#620755	see MEN1	yes				see MEN1	AD
VHL	von Hippel-Lindau syndrome	*VHL*	#193300	1. single gene sequencing 2. CNV analyses 3. multigene panel	VHL: 89%	VHL: 11%			broad clinical spectrum and overlap with several disorders.	AD
PPGL/PCC	Hereditary Paranglioma- Pheochromocytomas	*MAX*	#171300	multigene panel; for specific phenotypes: sequencing of *SDHB, SDHD*	dependent on the gene: up to 100%	up to 15%			Broad clinical spectrum and overlap with several disorders. It includes Cushing syndrome.	AD
		*SDHA*	#614165							AD
		*SDHAF2*	#601650							AD
		*SDHB*	#115310							AD
		*SDHC*	#605373							AD
		*SDHD*	#168000							AD, paternal inheritance
		*TMEM127*	#171300							AD
		*others*								
Growth, Obesity and Metabolism*										
NS	Noonan syndrome	*PTPN11*	#163950	(1. sequencing of PTPN11) **2. multigene panel**	nearly 100%				NS belongs to the group of RASopathies sharing affection of RAS pathway genes and overlapping features.	AD, rarely AR
		*SOS1*	#610733							
		*RAF1*	#611553							
		*RIT1*	#615355							
		*others*								
BWS	Beckwith-Wiedemann syndrome	11p15.5	#130650	1. methylation-specific test		< 1%		50%	Broad clinical spectrum and overlap with several disorders.	sporadic, rare cases: AD; somatic mosaicism
				2. *CDKN1C* testing	sporadic: 5% familial: 50%					AD, maternal inheritance
				3. multigene panel	single cases					AD, AR, X-linked
SRS	Silver-Russell syndrome	11p15.5	#180860	1. methylation-specific test				40%	Broad clinical spectrum and overlap with several disorders	sporadic, rare cases: AD; somatic mosaicism
				2. Microarray			10%			AD
				3. WES	up to 10%					AD, AR, X-linked
		7		methylation-specific test				10%		som. Mosaic
		14q32		methylation-specific test				10%		som. Mosaic
PWS	Prader-Willi syncdrome	15q11.2	#176270	CNV analyses		75%			Clinical overlap with several disorders	sporadic; rare cases: AD
				methylation-specific test (also detects 15q11.2 CNVs)		75–80%		20–25%		
IGHD	Isolated growth hormone deficiency type 1A	*GH1*	#262400	single gene sequencing	yes				Overlap with disorders caused by mutations in other members of the GH axis.	AR
	Isolated growth hormone deficiency type 1B	*GH1*	#612781							AR
	Isolated growth hormone deficiency type 2	*GH1*	#173100							AD
	Isolated growth hormone deficiency type 4	*GHRHR*	#618157	single gene sequencing	yes					AD
LS	Laron dwarfism	*GHR*	#262500	single gene sequencing	yes					AR
GHIP	partial growth hormone insensitivity / Increased responsivness to growth hormone		#604271							AD
IGF1 deficiency	IGF1 deficiency	*IGF1*	#608747	single gene sequencing	yes				see text	AR
IGF1RES	IGF1 resistancy	*IGF1R*	#270450	single gene sequencing	yes				see text	AD, AR
*Sex Development and Maturation*										
DSD	Disorders of sex development	*SRY, AR,*> 30 others		1. Cytogenetics (2. single gene sequencing) **3. multigene panel**	yes	yes	yes		broad clinical spectrum and overlap.	AD, AR, X-linked
TS / UTS	Turner syndrome	45,X		cytogenetics			100%		see text	de-novo
KS	Klinefelter syndrome	47,XXY		cytogenetics			100%		see text	de-novo

This position paper of the European reference networks on rare endocrine conditions (ENDO-ERN; www. https://endo-ern.eu) summarizes the current role of genetic testing in the diagnostic workup of (inherited) endocrine disorders and emphasizes the chances and advantages of modern genetic tests as well as the accompanying challenges and limitations. The paper will mainly refer to molecular germline variants and congenital disorders and will not cover genetic testing of somatic variants in tumors, which requires different considerations.

Due to the permanent improvement in diagnostic testing and increasing number of genetic factors associated with endocrine disorders, this review can only provide an overview on testing strategies and available tests. The authors therefore kindly ask the readers to visit curated and public databases like orphanet (https://www.orpha.net/consor/cgi-bin/Disease_Search.php?lng=EN) to get an up-dated overview on available diagnostic tests and therapies.

### Relevance of genetic testing for the management of endocrine disorders

The decision on the application of genetic tests is not only based on considerations about the benefit for the patient and therapeutic options which can be inferred from the genetic test result, but should also be based on the feasibility of genetic tests, their availability, informational value, and cost-effectiveness. It should be mentioned that predictive genetic testing in children without therapeutic consequences is not indicated and even violates the law in some countries.

**Fig. 1:**
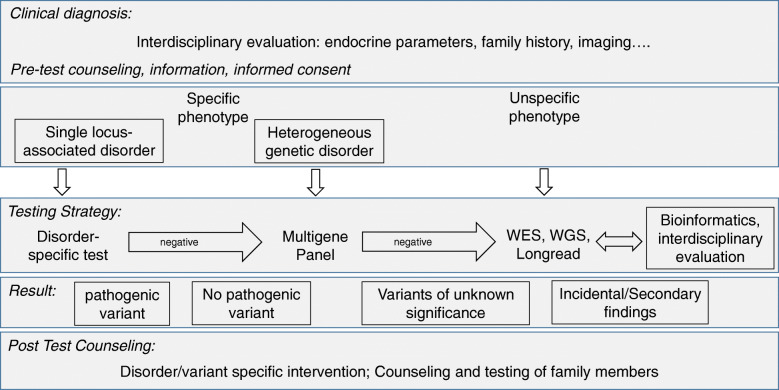
Molecular diagnostic workup in endocrine diseases. Genetic testing should be based on a comprehensive clinical diagnostic workup as a detailed phenotypic description both of clinical as well as endocrine laboratory features is key to the accuracy and yield of molecular testing. If possible, a targeted testing strategy should be preferred to avoid incidental findings. However, for very heterogeneous disorders WES-based approaches are suitable (for examples see Table 1)

The prerequisite of a targeted and efficient genetic test is the comprehensive evaluation of phenotype (deep phenotyping) and recording of the medical history by using a standardized and curated terminology (e.g. Human Phenotype Ontology (HPO)), which helps to specify the order for genetic testing (Fig. 1). In many instances, specialized endocrine diagnostic approaches with baseline and dynamic tests are required and may be supplemented by dedicated functional imaging procedures and specific hormonal analyses. Furthermore, precise molecular diagnosis may direct laboratory evaluation to “condition-specific target ranges” rather than to comparison to usual reference ranges [8] (Table 1).

An example for the need of precise clinical data as the basis for targeted genetic testing is short stature [9] where the first assessment comprises the analysis of growth parameters like height, weight and head circumference, as well as growth velocity and the skeletal features (bone deformities, demineralization, etc.). Endocrine tests (e.g. serum IGF1 concentration) may point towards the basic endocrinopathy and narrow down the specific defect, such as IGF1 deficiency or IGF1 resistance. Another example is the determination of Anti-Müllerian hormone (AMH) concentration in serum of patients suspected of Persistent Müllerian Duct Syndrome (PMDS), which identifies relevant genes to be sequenced: If serum AMH is undetectable the *AMH* gene should be sequenced, whereas analysis of the *AMH-R* gene is indicated in case AMH is normal/high in PMDS.

The central role of the precise molecular diagnosis as a decision aid for personalized clinical managements has meanwhile been shown for a broad range of endocrine disorders (Table 1), ranging from tumor predisposition syndromes [10] to disorders characterized by growth defects [9], glucose and insulin homeostasis (for review: [11]), obesity and lipodystrophy, hypogonadotropic hypogonadism [12], disorders of skeletal metabolism [13], and disorders of sexual development [14].

The therapeutic relevance of a precise genetic diagnosis can be illustrated for patients with growth disturbance disorders associated with molecular defects affecting the chromosomal region 11p15.5, i.e. Silver-Russell and Beckwith-Wiedemann syndrome (SRS, BWS). SRS is characterized by severe intrauterine and postnatal growth retardation, body asymmetry, feeding difficulties, relative macrocephaly at birth and characteristic facial features (for review: [15]). Among these features growth retardation is in the focus of the clinical management, and therapy is mainly based on recombinant growth hormone (rGH) treatment. The clinical heterogeneity results in an overlap with several other congenital growth retardation disorders and clinical misdiagnosis. These differential diagnoses comprise several tumor predisposition disorders (e.g. Bloom syndrome (OMIM #210900) or Mulibrey nanism (OMIM #253250)), for which rGH treatment is contraindicated [16]. In summary, the precise determination of the molecular cause of growth retardation in patients with SRS features is already nowadays required as the basis of a personalized therapeutic intervention (e.g. rGH treatment, tumor risk assessment). BWS is another example of an imprinting disorder associated with a broad spectrum of molecular alterations in 11p15.5. However, in contrast to SRS, BWS is characterized by overgrowth and an increased risk for embryonal tumors (for review: [17]). In fact, an association between specific molecular subtypes and tumor predisposition has been identified, and therefore the precise diagnosis of the molecular change in 11p15.5 has an impact on the tumor screening monitoring program [18]. Another example for the value of precise genetic subtyping for clinical decision-making is congenital hyperinsulinism due to K_ATP_ channel mutations, where biallellic mutations in either *ABCC8* or *KCNJ11* predict a diffuse type, while a single paternally inherited recessive mutation is highly suggestive of a focal type and may entail specific imaging and curative treatment (e.g. [11]).

Medical history should be accomplished by documentation of the family history of at least three generations and – if applicable – consanguinity and ethnicity. The family history might help to delineate the mode of inheritance and therefore provide hints at the disease-causing gene and mutation. However, the clinical manifestation and penetrance of genetic diseases can be highly variable even within the same family, therefore even minor clinical symptoms in apparently unaffected family members should also be checked thoroughly. Furthermore, there is a growing number of inherited disorders that do not exhibit the classical modes of inheritance, i.e. autosomal dominant, autosomal recessive or gonosomal inheritance (“Mendelian disorders”), but which differ from these rules. Mitochondrial inheritance is one example, as the respective disorders follow and exclusively maternal transmission pattern. Other examples of non-Mendelian inheritance are imprinting disorders like the Prader-Willi and Silver-Russell syndromes, in which the sex of the parent transmitting the molecular basic mechanism contributes to the phenotypic expression (for review: [19, 20]), as well as some trinucleotide disorders like the *FMR1*-associated premature ovarian failure and fragile X syndrome [21].

### Molecular alterations in endocrine disorders

Though the majority of pathogenic variants consist of pathogenic variants affecting only single nucleotides (single nucleotide variants, SNVs), there are further types of molecular alterations which can be associated with endocrine disorders (Table 1). SNVs as well as losses, gains or rearrangements (e.g. deletions, duplications, insertion-deletions / indel) of a small number of basepairs commonly have an impact only on a single gene, whereas larger copy number variants (CNVs) might have an effect on several genes. In addition to alterations of the DNA itself, modification of imprinted gene clusters can be altered. These epimutations can result in the disturbance of the fine-tuned monoallelic expression of imprinted genes which are expressed either from the maternal or the paternal gene copy.

In the majority of known inherited endocrine disorders, the variant is either inherited and follows a classical Mendelian trait (i.e. autosomal-recessive, autosomal-dominant, X-linked) or arises de-novo (in case of autosomal-dominant mutations). However, in the latter case these variants as well as epimutations might arise after fertilization, and can therefore occur as somatic mosaicism, meaning that not all cells of an organism carry the variant. In case of mosaicism, the ratio of cells with different (epi)genotypes can differ considerably, as demonstrated for Silver-Russell syndrome and McCune Albright syndrome (e.g. [22, 23])(Table 1). In some disorders, mosaicism is a well-known observation with a significant impact upon clinical manifestation and transmission risk (e.g. Neurofibromatosis type 1 [24]). As the presence of mosaicism can definitely not be excluded, testing of different tissues might be considered for every negative genetic test or particularly if an appropriate genetic test for a distinct phenotype comes back negative. Thus, the possibility of an undetected mosaicism should be discussed in a molecular genetic report if appropriate.

### Genetic tests and their applications in endocrine disorders

**Fig. 2:**
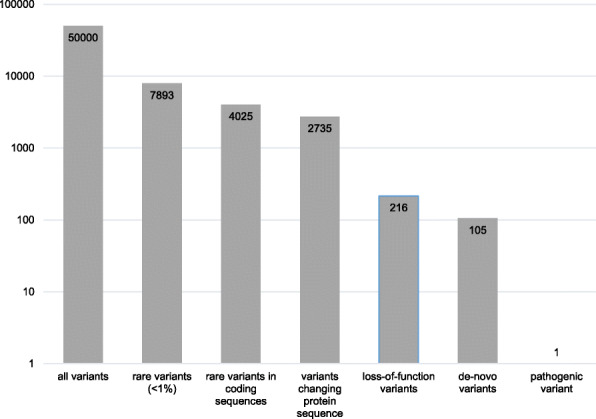
Example of filtering of genomic variants obtained by whole exome sequencing to identify a pathogenic variant in a growth retarded patientn. By applying different filter parameters like variant frequencies, pathogenicity and mode of inheritance, the number of genomic variants can be reduced and the disease-causing variant can be identified (numbers of variants are shown on the y axis)

Until recently, the detection of genomic variants of different sizes and nature often required the application of a step-wise process due to the limitations of the tests, accordingly this procedure was expensive and time-consuming. The parallel analysis of several genes, or even the comprehensive analysis of the whole genome by NGS, is a quantum jump in routine molecular diagnostics. In heterogeneous disorders with hundreds of genes known to cause similar and overlapping phenotypes (Table 1), these factors can now be analyzed within the same diagnostic run and assessment pipeline. Additionally, in case of NGS formats addressing the whole exome or the whole genome (WES: whole exome sequencing; WGS: whole genome sequencing), new genetic causes of diseases can be identified. Thus, the capability of genomic NGS is enormous, but in a diagnostic context it should be applied after estimating the advantages and disadvantages (Table 2). In fact, the estimation of the pathogenicity of genomic variants obtained by both WES and WGS even in protein-coding genomic regions is a major challenge. As illustrated in Fig. 2 for a patient with an unspecific growth retardation phenotype, WES results in a huge number (> 50,000) of genomic variants. As WGS addresses hundredfold of base pairs as many as WES, the number of genomic variants grows exponentially, and accordingly their interpretation might be extremely laborious. Thus, the bioinformatic pipelines need to become further automated to facilitate the interpretation of data.

**Table 2:** Currently applied methods in human genetic diagnostics of endocrine disorders: Applications, advantages and limitations. The methods can roughly be discriminated in respect to main type of molecular alteration they address, though some of them can also identify other changes. (*The currently used conventional diagnostic often address either copy number variants (CNVs, i.e. deletions and duplications) or single nucleotide variants (SNVs). In fact, CNVs represent a mutational burden in several genetic disorders. Therefore, parallel CNV assessment using alternate supplemental methods is normally required. For their identification, (semi)quantitative assays have been developed, and in human genetic testing multiplex ligation-dependent probe amplification (MLPA) is a broadly implemented diagnostic tool. However, the development of bioinformatics CNV pipelines for NGS data is in progress (e.g. [7]), and CNV detection by NGS is already in establishment. (*Multigene panels can either be based on targeted enrichment assays by which only the regions of interest are enriched in the wetlab, or they can be defined as a virtual WES dataset which has been filtered and analysed for the region of interest only. FISH: fluorescence in-situ hybridization, ASO: allele-specific oligonucleotide, MLPA: multiplex ligation-dependent probe amplification, SNP: single nucleotide polymorphism, CGH: comparative genome hybridization; WES: whole exome sequencing; WGS: whole genome sequencing; TGS: third generation sequencing; VUS: variant of unknown significance)

Method/Panel	Target region	Chances / Advantages	Limitations / Disadvantages
*Methods mainly addressing CNVs*			
Conventional cytogenetics	Whole genome	General overview on chromosomal number and structure; Mosaicism might be detected.	Resolution is > 5 Mb, smaller CNVs escape detection. SNVs not detectable. Cell culture required. Time and work consuming.
FISH	Specific chromosomal regions, whole chromosomes	Identification of structural rearragements. Detection of mosaicism.	Target region has to be known or should be suspected. Low resolution. Intact cells required.
Multiplex Ligation-dependent Probe Amplification (MLPA)	Single gene testing; specific genomic regions (60–100 bp)	Specific detection of genomic CNVs, appropriate for identification of deletions/duplications of selected exons.	Only targeted fragments are quantified. Restricted number of fragments per analysis (up to 60).
Whole genome imaging	Whole genome, specific chromosomal regions	General overview on chromosomal number and structure; Identification of structural rearrangements.	Detection of both numerical and structural aberrations with a relative high resolution (> 150 kb). Fresh samples required.
Microarray (SNP array, array CGH)	Whole genome	General overview on copy number variants, resolution of few kilobases.	Balanced chromosomal aberrations not detectable. Resolution on single gene level might be difficult.
NGS assays (Panels, WES, WGS, TGS)	See below	Comprehensive overview, dependent on the bioinformatics pipeline CNVs and structural variants can be detected	See below
*Methods/Panels mainly addressing SNVs*			
Single variant testing / Hotspot-mutation: e.g. ASO, single fragment sequencing, fragment analysis	SNVs, Trinucleotide repeat expansion	Very specific, fast, cheap.	Only single variants or trinucleotide repeats are addressed.
Single gene testing (e.g. Sanger sequencing)	Single genes	Target specific, appropriate and economic tool for monogenetic single locus disorders with characteristic clinical signs.	Large genes difficult to analyze. Not appropriate for heterogeneous disorders.
Multigene panel*	Genomic sequences (mainly coding regions and neighbored intronic regions) of selected genes associated with specific phenotypes	Target analyses of a group of genes associated with specific phenotypes. Low chance for incidental findings. Suitable for heterogeneous disorders with specific clinical features.	In case new genes are identified, adaption of a panel might be difficult or delayed in time. Variants in genes associated with overlapping phenotypes (differential diagnoses) might not be included in a panel. Non-coding regions are not covered.
Clinical exome	Coding and regulatory domains of all genes known to harbor clinically relevant variants	Analysis of a huge number of clinically relevant genes. Both disease-specific genes as well as differential diagnostic genes are analyzed. Suitable for disorders with unspecific clinical features	Increased probability to detect incidental findings. Increased probability for VUS. Fixed panel, new disease-associated genes are integrated after a delay. Non-coding regions are not covered.
Whole Exome sequencing/WES	Coding regions of ~ 19,000 protein coding genes (~ 180,000 exons); 1–2% of the human genome	All protein coding regions are covered. Identification of new disease-causing genes possible. Suitable for disorders with unspecific phenotypes	Detection of VUS and incidental findings probable. Non-coding regions are not covered. Analysis, interpretation and storage of large datasets required.
Whole Genome sequencing/WGS (short read)	Total human genome	Whole genome is analyzed. New genes as well as genomic variants in non-coding regions can be identified. Suitable for disorders with unspecific phenotypes.	Detection of VUS and incidental findings very probable. Analysis, interpretation and storage of very large datasets required.
Third Generation Sequencing (long read, TGS)	Ranging from defined chromosomal region to whole genome	Identification of chromosomal rearrangements and CNVs. Determination of physical breakpoints.	Resolution on single nucleotide level currently difficult.
*Methylation-specific testing*			
Single testing of imprinted loci (MS MLPA, MS pyrosequencing)	Single differentially methylated regions	Target specific, appropriate and economic tool for specific imprinting disorders.	Not appropriate for heterogeneous phenotypes. Multilocus disturbances are not detected.
Methylation-specific tests/Methylome	Ranging from single CpGs (e.g. PCR) and multilocus tests (e.g. MLPA) to genomewide analyses (array, NGS)	Identification of imbalanced methylation at selected CpGs. Different causes aberrant methylation pattern can be identified (UPD, CNV, epimutation). New and/or rare entities associated with disturbed imprinting can be identified.	Dependent on the test, different causes of aberrant methylation cannot be discriminated. In case of single and multilocus analyses non-targeted loci escape detection. In case of genome-wide analyses large datasets require comprehensive analyses and control data.
NGS assays: Panels, WES, WGS, TGS	See above	Comprehensive overview on altered methylation patterns.	See above
*Transcriptome*			
Transcriptome	Set of all RNA molecules in one cell or a population of cells	Identification of variants affecting splicing and causing allelic imbalances. Enhancement of the efficiency to identify functionally relevant variants. Complementary tool for WES and WGS.	Detected RNAs depend on the used tissues/cells. RNAs which are not expressed in this tissue are missed. Integration with data from other *omic* assays required

The decision on a genetic testing algorithm to confirm the clinical diagnosis of an endocrine disorder should consider both the range and types of pathogenic variants, as well as the appropriateness of the tests (Tables 1, 2):

In endocrine disorders presenting with characteristic phenotypic expression and caused by pathogenic variants in only one gene, like multiple endocrine neoplasia type 1 (*MEN1* gene) or von Hippel-Lindau syndrome (*VHL* gene), single gene testing is recommended. In some genetically heterogeneous disorders like Beckwith-Wiedemann syndrome and transient neonatal diabetes mellitus, a step-by-step molecular analysis might be meaningful, starting with the most frequent alterations. On the other hand, this stepwise strategy might not be useful in case of a suspected disorder with a genetically heterogenous background, variable expression and/or incomplete penetrance like permanent neonatal diabetes mellitus or hypothyroidism (for review: [25]). However, the causative genetic factors of several endocrine disorders can not be identified by these approaches, in these patients the application of WES and/or WGS should be considered. In fact, the deciphering of a disease-causing genetic change in these patients can be more successful if samples from the parents are also included in the analysis (trio-analysis).

In summary, specific tests targeting variants and genes will also be applied in the future in disorders with a small spectrum of mutations testing of which provides a high detection yield, whereas NGS based assays are used in genetically heterogeneous entities.

### Technical aspects of genetic testing in the NGS era

The chosen assay also influences the source and amount of the patients´ sample. For conventional cytogenetics, viable cells are required, whereas most molecular tests, including NGS, are based on genomic DNA which can be principally isolated from all freshly drawn or archived tissues. However, the molecular strategies also differ in the need of DNA.

The use of NGS gene panels allows to select and target specific groups of genes, and for some disorders it is therefore the first choice of testing. In contrast, NGS-based approaches covering hundreds of unselected genes or even the whole genome might lead to the inclusion of factors in the diagnostic setting that are functional candidates. The huge number of variants obtained by exome or genome-wide approaches reflect the variability and complexity of the human genome. In fact, the majority of variants represent non-pathogenic polymorphisms which also occur in control cohorts (Fig. 2). Thus, the key for a successful and efficient NGS data analysis is the stringent filtering by bioinformatic pipelines which commonly refer to (a) databases of genetic variants in humans, (b) pathogenicity prediction tools, and (c) different modes of genetic inheritance.

- With the increase of biological data ascertained by high-throughput omics technologies, the demand of databases on biological information has increased and the number of repositories is permanently growing (Nucleic Acids Research references 180 databases [26]). These curated databases daily exchange and update new data based on raw data from high-throughput laboratories.
- The freely or commercially available in-silico tools to predict the pathogenicity of a genomic variant mainly rely on its biochemical, structural, and functional properties, and its evolutionary conservation across species. Recent studies that compared the performance of the major prediction tools applied in genetic diagnostic testing have revealed a diverse picture of their reliability (for review: [27]). Therefore, the determination of pathogenicity should also consider additional information including variant frequencies obtainable from databases (see (a)), and segregation analyses in a family (see (c)), and – if possible – functional analysis.
- Segregation analysis seeking for the association of a genetic variant with the phenotype in a family is an appropriate tool to corroborate its pathogenicity.

The combination of these information should support the laboratory to delineate the pathogenicity of a genomic variant. With the guidelines for interpretation of genomic variants suggested by the American College of Medical Genetics [28], a widely accepted system for variant classification has been developed (Table 3). Whereas the classification of a variant as benign/likely benign or pathogenic/likely pathogenic either excludes or confirms its pathogenicity, the prediction of a considerable number of variants remains ambiguous (so-called variants of unknown significance – VUS). With the application of WES or WGS, the number of detectable variants including VUS grows exponentially, and NGS data therefore requires a stringent variant filtering (Fig. 2). These aspects should clearly be addressed before the application of such a broad test to avoid misunderstandings and unrealistic expectations, and the patients and their families should be informed about these scenarios with the help of appropriate patient counseling, information and informed consent forms [29]. However, the physician should be aware of the slight possibility that genomic variants which are classified as pathogenic/likely pathogenic at the time of diagnosis might be downgraded to benign later due to an increase of knowledge and datasets.

**Table 3:** Classification of genetic variants in routine diagnostics, leaned on the criteria suggested by the American College of Medical Genetics [28]

Clinical significance	Pathogenicity classes	Major Criteria
Clinical significance	Pathogenic Likely pathogenic	- The variant affects the structure and function of the gene/protein. - The variant affects a gene in which similar variants are known to be disease-causing. - The pathogenic nature of the variant is supported by epidemiological data, bioinformatic prediction and segregation analyses.
Uncertain significance	Variant of unknown significance (VUS)	- Not all parameters of pathogenicity are fulfilled. - Bioinformatics prediction of pathogenicity but without final confirmation.
No clinical significance	Likely benign Benign	- Epidemiological and bioinformatics data indicate that the variant is not pathogenic. - These variants are commonly not reported but might be available on request.

Another challenge in the diagnostic use of WES/WGS is the handling of incidental findings, i.e. genetic alterations associated with conditions or diseases unrelated to the patient’s present condition for which current tests are being performed but with important clinical ramifications. To circumvent these putative outcomes, which are difficult to manage in routine diagnostic workup and counseling, to reduce the costs for consumables and to avoid excessive amounts of data, targeted NGS panels have been established as an appropriate tool for NGS-based genetic testing (Table 1). However, multigene panels are not suitable for analysis of patients with unspecific phenotypes. In this situation the use of so-called “clinical exomes” might be discussed which target protein-coding regions of all genes for which disease-causing variants have been reported (e.g. “Kingsmore panel”). In fact, not all advantages and disadvantages of the different assay formats can be addressed in this paper, and every month new improvements of wet-lab and bioinformatic NGS tools as well as functional assays and suitable models to further characterize new variants are being published. For unusual phenotypes and challenging diagnostic scenarios, it is therefore recommended to contact laboratories experienced in NGS analysis in time for the up-to-date NGS testing strategies in connection with the pathology to be explored.

Laboratories offering genetic tests should implement a quality management system [30]. It should follow the national rules, but it should be leaned on the latest version of the ISO15189 standard, which specifies requirements for quality and competence in medical laboratories. Participation in external quality assessment schemes is a further key element of quality assurance in molecular genetic diagnostics, and these schemes help to test the laboratory workflow as well as the proficiency of data interpretation and reporting.

With the publication of guidelines for NGS testing, the European Society of Human Genetics has undertaken an essential step towards an international standard of NGS-based diagnostics [31].

## Conclusions

The implementation of NGS assays in DNA testing has significantly increased its diagnostic yield [4, 32], but it still leaves a considerable number of patients with an unusual clinical phenotype without molecular confirmation. With the rapid development of wet-lab assays and bioinformatic NGS pipelines it can be expected that the increasing application of NGS, as well as the improvements of databases and software tools underlying its data interpretation will significantly increase the rate of cases with a solved molecular basis. By complementing genomic NGS data with transcriptome (RNAseq) as well as methylome data and information from multiple *omic* sources, future diagnostic approaches will additionally become more comprehensive [33].

The growing knowledge on the contribution of genetic factors to endocrine disorders and the rapid implementation of new molecular tools in the diagnostic settings makes the interpretation of diagnostic data increasingly complex. Therefore, the data assessment should be embedded in interdisciplinary discussions using all available clinical and molecular information. Therefore, the metabolic and hormonal assessment remain fundamental. However, WES, WGS and further NGS formats are indispensable tools to identify new pathophysiological mechanisms of human disorders and to improve diagnostic algorithms. In the future, nearly all genetic alterations will be addressable by comprehensive NGS approaches.

Finally, the knowledge on the genetic cause of a disease does not only allow a precise clinical management, but it also helps to avoid invasive and expensive diagnostic tests which burden the patient, and lead to a faster diagnosis allowing an earlier and therefore more effective medical intervention (for review: [25]). The early diagnosis of a genetically based disorder supports the patients and their family in their self-determined planning of life as early as possible. Furthermore, it allows an accurate prognosis of recurrence risks for family planning as the basis of genetic counselling. Asymptomatic carriers of pathogenic variants can be identified, and prenatal testing might be offered, where appropriate.

## Data Availability

Not applicable.

## Abbreviations

AMH: Anti-Müllerian hormone
BWS: Beckwith-Wiedemann syndrome
CNV: Copy Number Variant
ENDO-ERN: European Reference Network on Rare Endocrine Conditions
HPO: Human Phenotype Ontology
PMDS: Persistent Müllerian Duct Syndrome
SNV: Single Nucleotide Variant
SRS: Silver-Russell syndrome
VUS: Variant of Unknown Significance
WES: Whole Exome Sequencing
WGS: Whole Genome Sequencing
